# Novel brain SPECT imaging unravels abnormal cerebral perfusion in patients with postural orthostatic tachycardia syndrome and cognitive dysfunction

**DOI:** 10.1038/s41598-025-87748-4

**Published:** 2025-01-28

**Authors:** Marie-Claire Seeley, Howard O’Brien, Gemma Wilson, Clair Coat, Tess Smith, Kevin Hickson, Reynold Casse, Amanda J. Page, Celine Gallagher, Dennis H. Lau

**Affiliations:** 1 Australian Dysautonomia and Arrhythmia Research Collaborative, Adelaide, SA Australia; 2 Faculty of Health and Medical Science, The University of Adelaide, Adelaide, SA Australia; 3 South Australian Health and Medical Research Institute, Adelaide, SA Australia; 4 Monash Health, Clayton, VIC Australia; 5 The Queen Elizabeth Hospital, Woodville, SA Australia; 6 Department of Cardiology, Royal Adelaide Hospital, 1 Port Road, Adelaide, SA 5000 Australia

**Keywords:** Postural orthostatic tachycardia syndrome, Cerebral blood flow, Brain SPECT, Orthostatic intolerance, Post-acute sequelae of COVID-19, Cardiology, Neurology

## Abstract

Cognitive dysfunction is frequently reported in individuals with postural orthostatic tachycardia syndrome (POTS), possibly resulting from reduced cerebral blood flow (CBF). We used brain SPECT, an accessible imaging modality that has not been systematically evaluated in this patient group. Retrospective review of participants from our registry was undertaken to identify those who had a brain SPECT performed for investigation of cognitive dysfunction. Abnormal CBF was taken as z-score > 2 standard deviations of healthy control reference values. Patient reported outcome measures (PROMs) such as autonomic, gastric and quality of life symptom scores were analyzed. From a total of 56 participants (mean 34.8 ± 10.7 years, 88% females), PROMs indicate: moderate to severe autonomic dysfunction in 75%; at least mild to moderate gastroparesis in 23%; low global health rating and utility scores. Abnormal CBF was seen in 61% but did not differ by POTS triggers. The regions with the lowest mean z-scores were the lateral prefrontal and sensorimotor cortices. Hierarchal regression analyses found number of brain regions with abnormal CBF, autonomic and gastric symptoms to account for 51% of variances in health utility. Cerebral hypoperfusion is prevalent in those with POTS and cognitive dysfunction even whilst supine, contributing to reduced quality of life.

## Introduction

Postural orthostatic tachycardia syndrome (POTS) is a heterogenous autonomic disorder which manifests in multisystemic symptoms, including postural induced tachycardia in the absence of orthostatic hypotension^1,2^. The condition is associated with high health disutility and low health related quality of life (HrQoL) as well as high female and Caucasian preponderance^2–5^. Temporal association with viral infection is frequently reported and more recently POTS has been identified as the dominant phenotype in post-acute sequelae of COVID-19 (PASC)^6,7^. The pathogenesis of POTS remains poorly understood with purported mechanisms including increased sympathetic activity, excessive catecholamine, impaired vagal activity, peripheral autonomic neuropathy, abnormal G-protein coupled receptor autoantibody and hypovolemia^8,9^. In addition to viral insult, trauma, pregnancy and vaccination are also reported to be triggers for POTS^2^.

POTS continues to be poorly recognized among clinicians which is evidenced by the often-reported protracted delay in diagnosis^5,10^. Amidst a wide spectrum of symptoms, patients describe experiencing neurocognitive dysfunction, characterized by diminished concentration, impaired memory, and slowed thinking processes, commonly referred to as ‘brain fog’^2,10,11^. Previous transcranial Doppler studies have found reduced cerebral blood flow (CBF) in those with POTS during orthostatic and cognitive stress^11–14^. However, the Doppler technique remains confined to research settings due to the expertise and time required for measurements and potential inter-operator variability^15^. On the other hand, single photon emission computed tomography (SPECT) is a broadly accessible technology which provides an indirect marker of functional changes and can be used as an indication of CBF^16^. SPECT imaging has been shown to detect cerebral hypoperfusion even when computed tomography and magnetic resonance imaging (MRI) findings are normal. Despite these modalities and positron emission tomography can offer superior spatial resolution, their limited accessibility and higher costs restrict their broad clinical utility. Additionally, nuanced differences exist between positron emission tomography and SPECT, with the former detecting metabolism and the latter measuring cerebral perfusion^17,18^.

Brain SPECT has been shown to correlate clinically with cognitive and neuropsychological changes, highlighting its sensitivity to early neurogenic alterations in conditions such as traumatic brain injury and dementia^19,20^. Nevertheless, there remains a paucity of data relating to the use of SPECT in autonomic disorders such as POTS. Here, we present the first characterization of brain SPECT imaging in a POTS cohort. Our aim was to explore the spectrum of cerebral perfusion in POTS with a view to delineate possible associations between cerebral hypoperfusion in POTS with disease trigger, autonomic symptom burden and HrQoL.

## Results

### Participant characteristics

A total of 62 patients with POTS were identified to have previously undergone brain SPECT imaging from 440 patients enrolled in the registry over the study period. Six patients were excluded due to incomplete data leaving 56 participants eligible for inclusion in this analysis (Table 1 & Supplementary Fig. 2). The mean age was 34.8 ± 10.7 years with an average body mass index of 26.5 ± 6.4 kg/m^2^ and there was a Caucasian (95%) and female sex (88%) preponderance. Most of the participants were tertiary educated (71%), however, only 21% were in fulltime employment. The onset of POTS symptoms was reported at a median age of 28 years (IQR 16–37) after a diagnostic delay of 4.9 ± 6.1 years. A discernible trigger with temporal proximity to POTS symptom onset was found in 71% of the cohort. SARS-CoV-2 infection was the most common of these (29%) followed by infections other than COVID-19 (20%), and trauma/concussion (13%). A further 10% had a mixture of other associated onset triggers such as vaccination, pregnancy and surgery. Of the 16 patients with COVID-19 related POTS, 87% had at least two SARS-CoV-2 vaccinations prior to their infection. All had laboratory confirmed mild COVID-19 infection that did not require hospitalization or oxygen therapy. The most common co-morbid conditions were migraine (54%), hEDS (45%) and myalgic encephalomyelitis (36%).

**Table 1:** Patient characteristics.

	Whole Cohort (n = 56)	Normal CBF (n = 22)	Abnormal CBF (n = 34)	p-value
Age (years), mean ± SD	34.8 ± 10.7	34.7 ± 11.7	34.9 ± 10.2	.931
Body mass index, kg/m^2^, mean ± SD	26.5 ± 6.4	25.4 ± 6.3	27.2 ± 6.4	.299
Female, n (%)	49 (87.5)	20 (91)	29 (85)	.428
Caucasian race, n (%)	53 (95)	21 (95.5)	32 (94.1)	.661
Tertiary educated, n (%)	40 (71)	15 (68.2)	25 (73.5)	.445
Full-time employment, n (%)	12 (21)	3 (13.6)	9 (16.1)	.211
Age of symptom onset (years), median (IQR)	28 (16, 37)	26 (19, 37)	28 (15, 37)	.830
Diagnostic delay (years), mean ± SD	4.9 ± 6.1	5.2 ± 5.7	4.7 ± 6.5	.736
POTS triggers, n (%)				
COVID-19 infection	16 (28.6)	4 (18.2)	12 (35.3)	.168
No identifiable cause	16 (28.6)	8 (36.4)	8 (23.5)	.298
Other viral infections	11 (19.6)	2 (9.1)	9 (26.5)	.110
Post trauma/concussion	7 (12.5)	3 (13.6)	4 (11.8)	.834
Comorbid diagnosis, n (%)				
Migraine	30 (53.6)	14 (63.6)	16 (47.1)	.174
Hypermobile Ehlers Danlos syndrome	25 (44.6)	7 (31.8)	18 (52.9)	**.043**
Myalgic encephalomyelitis	20 (35.7)	12 (54.5)	8 (23.5)	**.019**

Diagnostic delay – Time from symptom onset until diagnosis of POTS.

### Cerebral perfusion

**Fig. 1:**
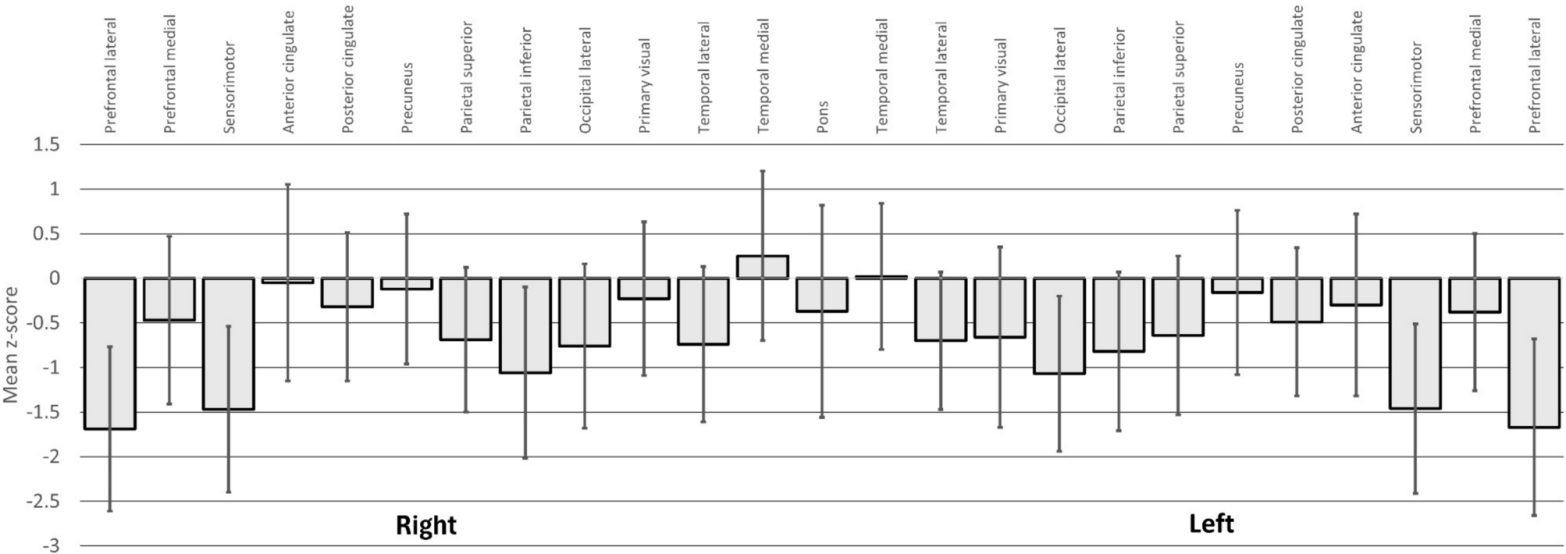
Mean z-scores of CBF by region. The means and standard deviations of the z-scores by region are shown from the left and right hemisphere.

In total, 61% (n = 34) of POTS patients demonstrated abnormal CBF on brain SPECT with 23 participants having at least three brain regions with z-scores less than two standard deviations of normal. There were no differences in patient demographics between those with normal and abnormal CBF (Table 1). Notably, the proportion of patients with hEDS was higher while the proportion of patients with myalgic encephalomyelitis was lower in the abnormal CBF cohort (Table 1). Amongst those with PASC related POTS, 75% demonstrated abnormal CBF. The regions with the most prevalent abnormal CBF were the left and right lateral prefrontal and left and right sensorimotor cortices (41.1%, 33.9%, 32.1%, 26.8% respectively, Table 2), followed by the pons and right inferior parietal cortex (17.9% & 14.3% respectively, Table 2). Mean z-scores were below zero for all regions studied except for the right and left temporal medial cortices (Fig. 1). The mean z-score (Table 2) was also lowest in the same regions: left and right lateral prefrontal (− 1.67 ± 0.99 and − 1.69 ± 0.92); left and right sensorimotor (− 1.46 ± 0.95 and − 1.47 ± 0.93); right inferior parietal cortex (− 1.06 ± 0.96); except for the pons (− 0.37 ± 1.19).

**Table 2:** Prevalence of abnormal CBF and mean z-scores by region.

	Abnormal CBF n (%)	z-scores mean ± SD
Prefrontal lateral R)	19 (33.9)	− 1.69 ± 0.92
Prefrontal lateral L)	23 (41.1)	− 1.67 ± 0.99
Prefrontal medial R)	2 (3.6)	− 0.47 ± 0.94
Prefrontal medial L)	1 (1.8)	− 0.38 ± 0.88
Sensorimotor R)	15 (26.8)	− 1.47 ± 0.93
Sensorimotor L)	18 (32.1)	− 1.46 ± 0.95
Anterior cingulate R)	3 (5.4)	− 0.05 ± 1.10
Anterior cingulate L)	1 (1.8)	− 0.30 ± 1.02
Posterior cingulate R)	2 (3.6)	− 0.32 ± 0.83
Posterior cingulate L)	3 (5.4)	− 0.49 ± 0.83
Precuneus R)	–	− 0.12 ± 0.84
Precuneus L)	1 (1.8)	− 0.16 ± 0.92
Parietal superior R)	2 (3.6)	− 0.69 ± 0.81
Parietal superior L)	3 (5.4)	− 0.64 ± 0.89
Parietal inferior R)	8 (14.3)	− 1.06 ± 0.96
Parietal inferior L)	4 (7.1)	− 0.82 ± 0.89
Occipital lateral R)	3 (5.4)	− 0.76 ± 0.92
Occipital lateral L)	6 (10.7)	− 1.07 ± 0.87
Primary visual R)	2 (3.6)	− 0.23 ± 0.86
Primary visual L)	6 (10.7)	− 0.66 ± 1.01
Temporal lateral R)	3 (5.4)	− 0.74 ± 0.87
Temporal lateral L)	1 (1.8)	− 0.70 ± 0.77
Temporal medial R)	3 (5.4)	0.25 ± 0.95
Temporal medial L)	1 (1.8)	0.02 ± 0.82
Pons	10 (17.9)	− 0.37 ± 1.19

R, denoted right; L, denotes left.

### Patient-reported outcome measures

Mean total COMPASS-31 score for the entire cohort was 49.6 ± 14.2, with 75% of participants having a total score ≥ 40 to indicate a moderate to severe burden of autonomic dysfunction. However, there were no differences in total COMPASS-31 score or individual subdomain score between those with normal or abnormal CBF (Table 3). Mean GCSI score for the entire cohort was 1.4 ± 0.75 with 23% of participants having GCSI score of ≥ 2 to indicate at least mild to moderate symptoms of gastroparesis. Similarly, there were no differences in total GCSI score or individual sub-score between those with normal or abnormal CBF (Table 3). According to the EQ-5D-5L scores for the entire cohort, moderate to extreme problems were encountered most frequently with ‘usual activities’ followed by 'pain and discomfort’, 'mobility’, ‘anxiety and depression’ and ‘self-care’ (84, 66, 52, 41 & 25%; Table 3). Notably, the median global health rating (EQ-VAS) was low amongst the entire cohort (31, IQR 25–49), as was the calculated mean EQ health utility score (0.53** ± **0.25). Although the EQ-VAS and EQ health utility score did not differ amongst those with normal and abnormal CBF, the cohort with abnormal CBF had higher rates of moderate to extreme problems with mobility (65 vs 32%;* P* = 0.016) and carrying out ‘usual activities’ (94 vs 68%; *P* = 0.014).

**Table 3:** Between group differences in symptom scores between those with and without rdCBF.

	Whole Cohort (*n* = 56)	Normal CBF (*n* = 22)	Abnormal CBF (*n* = 34)	p-value
Composite autonomic symptoms score-31, mean ± SD				
Orthostatic Intolerance	25.7 ± 7.4	24.2 ± 5.6	26.7 ± 8.2	*.212*
Vasomotor	2.4 ± 1.7	2.5 ± 1.6	2.3 ± 1.9	.748
Secretory motor	6.1 ± 3.8	5.7 ± 3.3	6.3 ± 4.2	.600
Gastrointestinal	10.1 ± 4.1	10.6 ± 3.8	11.1 ± 4.2	.621
Bladder	1.6 ± 1.9	1.8 ± 2.3	1.5 ± 1.6	.510
Pupillary motor	3.1 ± .9	3.0 ± .8	3.0 ± 1.1	.874
Total COMPASS-31 Score	49.6 ± 14.2	47.7 ± 11.1	50.9 ± 2.0	.430
Gastroparesis cardinal symptom index score, mean ± SD				
Nausea/vomiting	.81 ± .71	.9 ± .8	.77 ± .7	.597
Early satiety/post-prandial fullness	1.6 ± 1.1	1.5 ± 1.0	1.7 ± 1.1	.384
Bloating	1.8 ± 1.1	1.5 ± 1.0	2.0 ± 1.1	.099
Total GCSI score	1.4 ± .75	1.3 ± 0.8	1.5 ± .7	.315
GCSI score ≥ 2, n (%)	13 (23.2)	3 (13.6)	10 (29.4)	.149
EuroQol 5-Dimension-5L				
EQ-VAS, median (IQR)	31 (25,49)	30 (27, 50)	35 (23, 41)	.667
EQ health utility score, mean ± SD	.53 ± .25	.56 ± .25	.51 ± .25	.410
EQ-Subdomains, moderate to extreme problems n (%)				
Mobility	29 (51.8)	7 (31.8)	22 (64.7)	**.016**
Self-care	14 (25.0)	6 (27.3)	8 (23.5)	.495
Usual activities	47 (83.9)	15 (68.2)	32 (94.1)	**.014**
Pain and discomfort	37 (66.1)	13 (59.1)	24 (70.6)	.274
Anxiety and depression	23 (41.1)	9 (40.9)	14 (41.2)	.603

EQ-VAS, EuroQol visual analogue scale is a global health rating from 0 to 100 with ‘100’ = to full health; EQ-Utility index, EuroQol utility scale which rates health utility from 0–1 with ‘1’ = full health.

### Predictors of poor health utility

Hierarchical multiple regression analyses found three significant predictors of reduced health utility. After controlling for age and the multimorbidity, the forwards stepwise regression model found no significant predictive influence of anxiety, depression, hEDS, myalgic encephalomyelitis, migraine, COVID-19 trigger, or body mass index on health utility. In step one, age and multimorbidity explained 20% of the variances in the EQ utility score (*P* = 0.002). After adding the mean number of brain regions with abnormal CBF as well as the total COMPASS-31 and GCSI scores, the model as a whole, explained 51% of the total variances (*F* [5, 50]  = 10.24, *P* < 0.001). The added measures together explained an additional 30% of variances in EQ-utility scores (*R* squared change = 0.30, *F* change (3, 50) = 10.21, *P* < 0.001).

## Discussion

**Fig. 2:**
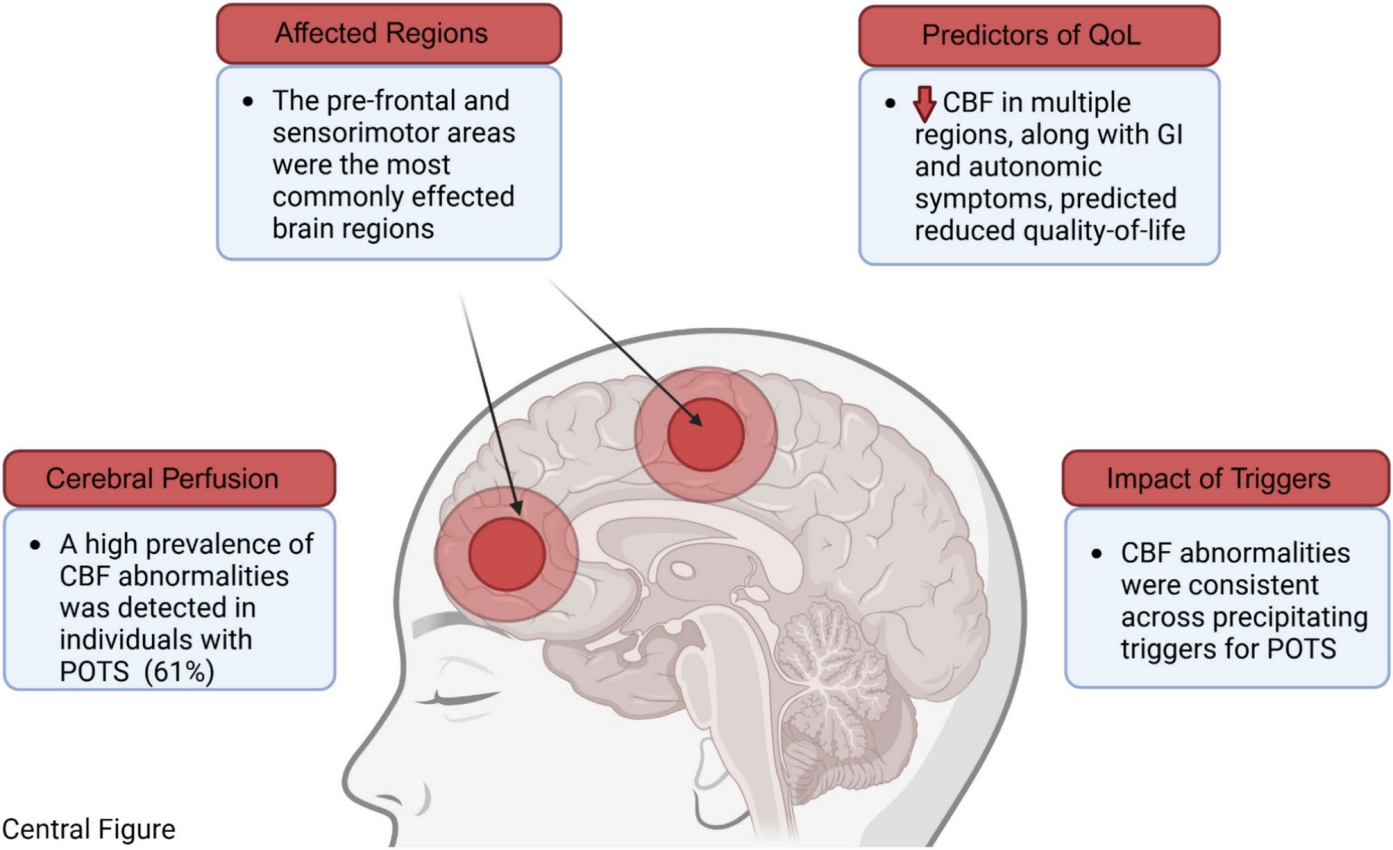
Cerebral blood flow abnormalities in POTS assessed by brain SPECT. This figure was created in https://BioRender.com.

This is the first study to describe abnormal cerebral perfusion using brain SPECT in a population with POTS demonstrating several findings of importance (Fig. 2). First, there was a marked prevalence of cerebral hypoperfusion in those with POTS and symptoms of neurocognitive dysfunction. Second, the pre-frontal and sensorimotor regions were the most affected cerebral regions. Third, an increased number of cerebral regions affected by perfusion abnormalities combined with worse gastrointestinal and autonomic symptoms were predictive of reduced quality of life. Last, the prevalence of abnormal CBF did not differ based on the precipitating trigger for POTS.

In our cohort, a strikingly high prevalence of cerebral hypoperfusion (61%) was observed, which aligns with the frequent reporting of neurocognitive symptoms in individuals with POTS^21^. Particularly noteworthy were the frequent occurrences of prefrontal lateral and sensorimotor regional hypoperfusion, areas known for their involvement in executive function and proprioceptive sensory and motor function respectively. This observation suggests a potential link between regional cerebral hypoperfusion and the reported symptoms of impairments in planning, organization, memory and attention, as well as pain and proprioceptive abnormalities in those with POTS^22^. Previous studies have delved into how both orthostatic and cognitive stress can impact cerebral perfusion in individuals with POTS, revealing a negative influence^11–13^. It is therefore likely that the abnormal CBF seen in our study would be more exaggerated in these situations as our brain SPECT imaging was undertaken in the supine position and at rest. Historically, transcranial Doppler and near-infrared spectroscopy have been favored for assessing CBF in POTS due to their flexibility during head-up tilt testing^15,23^. However, their clinical utility is limited either by the operational expertise required or restricted accessibility outside specialized research settings. In contrast, SPECT is more widely accessible to primary care physicians, allowing for the detection of cerebral hypoperfusion in POTS in the absence of the need to undertake imaging during orthostatic stress.

The similarities of symptoms in those with POTS and PASC are increasingly recognized, with some suggesting that the former represents the predominant autonomic phenotype of the latter^24–26^. PASC related POTS accounts for 29% of our cohort and we found no statistical difference in POTS triggers in those with normal versus abnormal CBF despite evidence of abnormal CBF in 75% of those with PASC related POTS. Interestingly, others have used various imaging techniques to evaluate neurocognitive dysfunction including F-Fluorodeoxyglucose positron emission tomography (F-FDG-PET), functional MRI, diffusion MRI, and SPECT in those with PASC^27^. They identified a high prevalence of functional imaging abnormalities in PASC with findings of cerebral hypoperfusion that correlated with functional decline^27^. Our findings are similar to those reported in the PASC population although our patients with POTS had an average symptom duration of 6 years prior to their brain SPECT while those with PASC in our cohort were studied at almost 1 year since their acute COVID-19 infection. It appears that abnormal CBF may persist substantially longer in those with POTS and may not be a unique functional marker due to PASC.

Others have shown that those with joint hypermobility and myalgic encephalomyelitis had more pronounced reduction in CBF on transcranial doppler during orthostatic stress than those without joint hypermobility^28^. In our study, hEDS was similarly associated with higher rates of abnormal CBF on SPECT, highlighting the need for further investigations into the interrelated effects of these disorders and neurocognitive impairment. It is noteworthy that hEDS exhibits a high concurrence with POTS with up to 30% of patients meeting diagnostic criteria for both conditions and the reduced venous return due to vascular laxity and blood pooling may be responsible for reduced CBF in this population^29^. In contrast, abnormal CBF was seen in only 40% of our cohort with myalgic encephalomyelitis despite higher prevalence of 80% in a previous study of 60 subjects with cortical hypoperfusion seen at frontal, parietal, temporal and occipital areas^30^. CBF may be more impaired in the setting of orthostasis with Doppler evidence of increasing neurocognitive impairment and uncoupling of the neurovascular unit with increasing tilt angle in those with myalgic encephalomyelitis^11^. The disparity of findings may be due to the small sample size in our cohort.

Individuals with abnormal CBF in our study had significantly more problems with their mobility and carrying out of usual activities than those without abnormal CBF. Importantly, our study revealed that an increased number of cerebral regions affected by perfusion abnormalities, combined with worsened gastrointestinal and autonomic symptoms, were predictive of reduced quality of life and functional impairment, emphasizing the clinical relevance of these interrelated factors in shaping patient outcomes. Considering the young age of our POTS cohort, predominantly comprising females, and the concerning implications of cerebral hypoperfusion on cognitive function and motor control, our findings raise questions about the potential impact on educational progression, workforce engagement, and overall social and economic well-being. Notably, the overall cohort’s mean health utility score was low at 0.53 and it is at a level below that of other chronic health conditions such as chronic renal failure, diabetes and cardiovascular disease^2^. Further studies are warranted to elucidate whether cerebral perfusion abnormalities persist longitudinally in POTS and whether specific treatments may improve cerebral perfusion on SPECT and HrQoL.

The cross-sectional, retrospective, and single-center design of this study limits the generalizability of our results. Brain SPECT imaging was undertaken for clinical investigation of neurocognitive impairment and without formal cognitive assessments to allow for clinico-radiological correlation. This highlights the need for future longitudinal research incorporating standardized cognitive testing to explore this association further. While CBF abnormalities were determined based on quantification using z-scores, these scores may not directly correlate with clinical symptomatology and subtle deviations from the cut-offs may still be functionally or clinically relevant. Consequently, this study may underestimate the prevalence of clinically relevant cerebral hypoperfusion in POTS. The absence of comparative structural and functional brain imaging limited our ability to delineate the neurobiological underpinnings of cerebral hypoperfusion. Additionally, the use of the proprietary control database, as well as the retrospective nature of the study precluded the use of a study-specific control group. To address these limitations, prospective, studies utilising well-characterised control cohorts with transparent demographic and sample size data are needed to validate these findings. Longitudinal studies are needed to assess if interventions targeting autonomic dysfunction can improve cerebral perfusion. Despite these limitations, the notable presence of cerebral hypoperfusion observed in our cohort underscores the necessity for future robust prospective studies to comprehensively characterize cerebral perfusion in autonomic disorders and assess its clinical utility.

## Conclusions

There is a high prevalence of reduced cerebral perfusion in those with POTS, which is detectable with supine brain SPECT imaging and is associated with diminished quality of life. The most frequently affected areas are prefrontal lateral and sensorimotor cortices. Reduced cerebral perfusion appears to be more prevalent in those with comorbid joint hypermobility syndrome and independent of POTS triggers. These results highlight the need for further research to delineate the etiology and mechanistic pathways underlying neurovascular dysfunction in post-viral and autonomic syndromes including long-COVID. Future prospective studies may explore the role of therapies such as exercise, vasoconstrictors and fluid management on cerebral perfusion.

## Methods

A retrospective audit of participants ≥ 16 years of age and enrolled in the Australian POTS patient registry (Australian New Zealand Clinical Trials Registry: ACTRN12621001034820) between 5th May 2021 and 7th August 2023 was undertaken to identify those with a brain SPECT performed for clinical investigation of neurocognitive dysfunction or brain fog. All participants gave informed consent prior to their inclusion in the registry. The study was performed in accordance with the 1964 Declaration of Helsinki standards for human research with institutional ethics board approval (H-2021-052). To be eligible for inclusion in the patient registry, all participants had clinical diagnosis of POTS according to the accepted international criteria with: a sustained heart rate rise of ≥ 30 bpm on a 10-min head up tilt or active standing test; absence of orthostatic hypotension as defined by a drop of ≥ 20 mmHg systolic or 10 mmHg diastolic blood pressure in the first three minutes of standing; and symptoms of orthostatic intolerance for ≥ 3 months^1^.

Additionally, inclusion in this retrospective review required completion of symptom and HrQoL surveys, including the EuroQol 5 Dimension-5L (EQ-5D-5L), composite autonomic symptom score (COMPASS-31), and gastroparesis cardinal symptom index score (GCSI), via a password protected research electronic data capture (REDCap) database. In brief, the 5-response version of the Euroqol 5 Dimension questionnaire is a non-disease specific instrument that demonstrates strong sensitivity in detecting clinically meaningful differences in HrQoL across five domains: mobility, self-care, usual activities, pain/discomfort and anxiety/depression^31^. Respondents were asked to rate from no problems (1) through to extreme problems (5) on a Likert scale for each domain and provide a global health rating on a visual analogue scale (EQ-VAS) from 0 to 100 (with 100 equal to a state of full health)^31^. The Devlin et al. (2016) UK data set was utilized to calculate a utility score on a scale where ‘1’ equals to full health and ‘0’ equals a state commensurate with death^31,32^. The COMPASS-31 assesses autonomic dysfunction across six domains of orthostatic intolerance, vasomotor, secretory motor, bladder, gastrointestinal and pupillary motor function with higher overall score representing worse autonomic dysfunction^33^. Participants rated their gastrointestinal symptoms on a scale of 1 to 4 (mild to severe) across three sub-domains of GCSI: post-prandial fullness/early satiety, nausea/vomiting, and bloating. A GCSI score of ≥ 2 indicated at least mild to moderate symptoms of gastroparesis^34^. Routine clinical assessment for hypermobile Ehlers Danlos Syndrome (hEDS) and hypermobile spectrum disorder was undertaken at the time of POTS diagnosis using the 2017 hEDS diagnostic checklist^35^. In short, the checklist uses three criteria 1. generalized joint hypermobility, 2. systemic features and 3. exclusionary criteria, to diagnose hEDS. For the purposes of analysis, multimorbidity was defined as concomitant diagnosis of three or more chronic illnesses.

### Brain SPECT

Referral for brain SPECT imaging was based on the clinical judgement of independent treating physicians for symptoms of neurocognitive dysfunction. Standard patient preparation includes abstinence from smoking and alcohol for 10 h prior to imaging to limit extraneous attenuating alterations to cerebral perfusion. All SPECT/CT images of the brain were obtained following intravenous administration of Tc99m-hexamethylpropylene-amine oxime (HMPAO) tracer, which was injected under resting conditions in a quiet, dimly lit room. Patients were injected with 750 MBq(+ /− 10%) tracer in a volume of 0.4–0.5ml. Fifteen minutes after radiopharmaceutical injection, low energy high resolution brain SPECT scan was acquired using either the GE NM/CT Discovery 670 or NM/CT Discovery 870 (GE Healthcare, Boston, MA, USA). The SPECT was acquired in step and shoot mode with 3° rotation, 120 views and 30 s pre-step. Each system has a 16-slice CT with acquisition parameters of 120 kV and 20 mA and attenuation correction for the density of the surrounding tissue. Cortical distribution of radiotracer was assessed for symmetrical perfusion.

Quantitative SPECT analysis using proprietary third-party software has been shown to be compatible with, and in some cases superior to, qualitative reporting and lends itself more readily to quantitative analysis^36^. The location of a significant regional decrease in cerebral blood flow for SPECT was analyzed using a statistical quantitative brain mapping technology (3D-SSP/Neurostat, The University of Utah, Salt Lake City, AZ, USA)^37^ and compared with Q.Brain on the Xeleris 4 Workstation (GE Healthcare) to ensure consistency. Q.Brain and Neurostat derive z-scores by comparing a patient’s brain imaging data to a normative database of healthy individuals. This involves spatially normalizing the images to a standard atlas, then performing voxel-wise comparisons to calculate z-scores, which indicate the number of standard deviations a patient’s cerebral blood flow deviates from the normative database of healthy controls. Z-scores ≥ 2 standard deviations were taken to represent abnormal perfusion as per previous validation^38^. As 3D-SSP/Neurostat and Q.Brain exhibited similar activity distribution, regional z-scores derived from an age-matched normal control database from Q.Brain were used for cerebral blood flow (CBF) analysis (representative outputs are shown in Supplemental Figure 1). All results were qualitatively corroborated and adjudicated by the on-duty consultant nuclear medicine physician.

### Statistical analysis

Categorical variables were described as frequency or percentages, while continuous data were expressed as mean and standard deviation or median and interquartile range (IQR) according to distribution. The Mann–Whitney U Test (two groups) and Kruskal–Wallis one-way analysis of variance (multiple groups) were used for continuous data while the Chi-squared test was used for categorical data. A hierarchical forwards stepwise regression analysis was undertaken to explore predictors of reduced quality of life as indicated by the EQ utility score. A minimally adjusted model was performed to control for age, and multimorbidity which have previously been shown to reduce quality of life^31^. The following variables were explored in a step by step fashion: PASC diagnosis, autonomic, and gastric symptom scores as well as the number of brain regions with abnormal CBF. The final fully adjusted model included those variables found to have a significant predicting effect on EQ utility scores. All data were analyzed using SPSS statistics (version 28.0, IBM Inc, Armonk, NY, USA) and statistical significance was set at p < 0.05.

## Supplementary Information

Supplementary Information.

## Data Availability

De-identified data from this study are available from the corresponding author upon request.
