# Supplementary material for: Novel brain SPECT imaging unravels abnormal cerebral perfusion in patients with postural orthostatic tachycardia syndrome and cognitive dysfunction
Source: Sci Rep. 2025 Jan 28;15:3487. doi: 10.1038/s41598-025-87748-4 (PMC11775248; doi:10.1038/s41598-025-87748-4)
Supplement: Supplementary file 1 — Supplementary Information. [file 41598_2025_87748_MOESM1_ESM.docx]

**Supplementary Figure 1A: Brain SPECT image demonstrating prefrontal, sensory motor and left parietal hypoperfusion in a POTS patient**


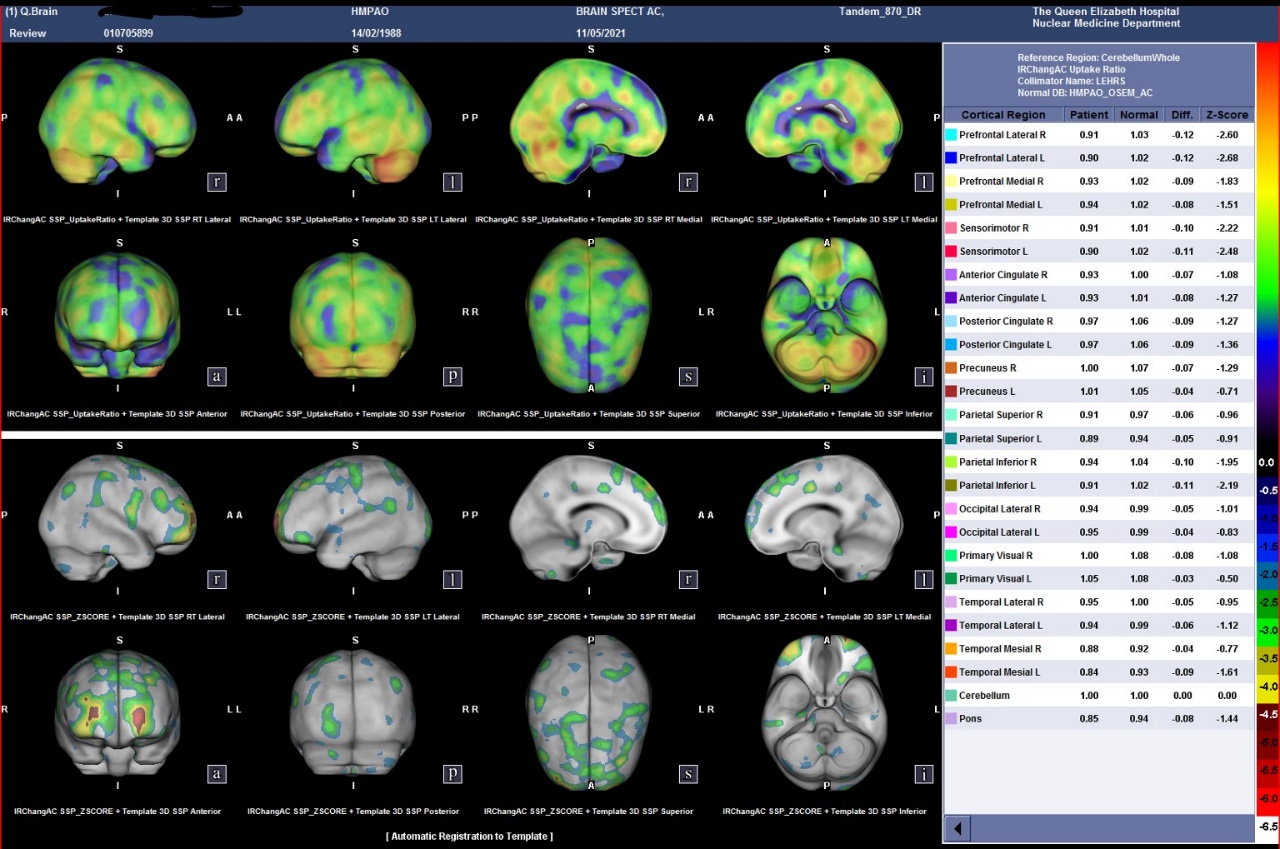


**Supplementary Figure 1B. Brain SPECT image demonstrating normal cerebral perfusion in a 33 year old non-POTS patient**


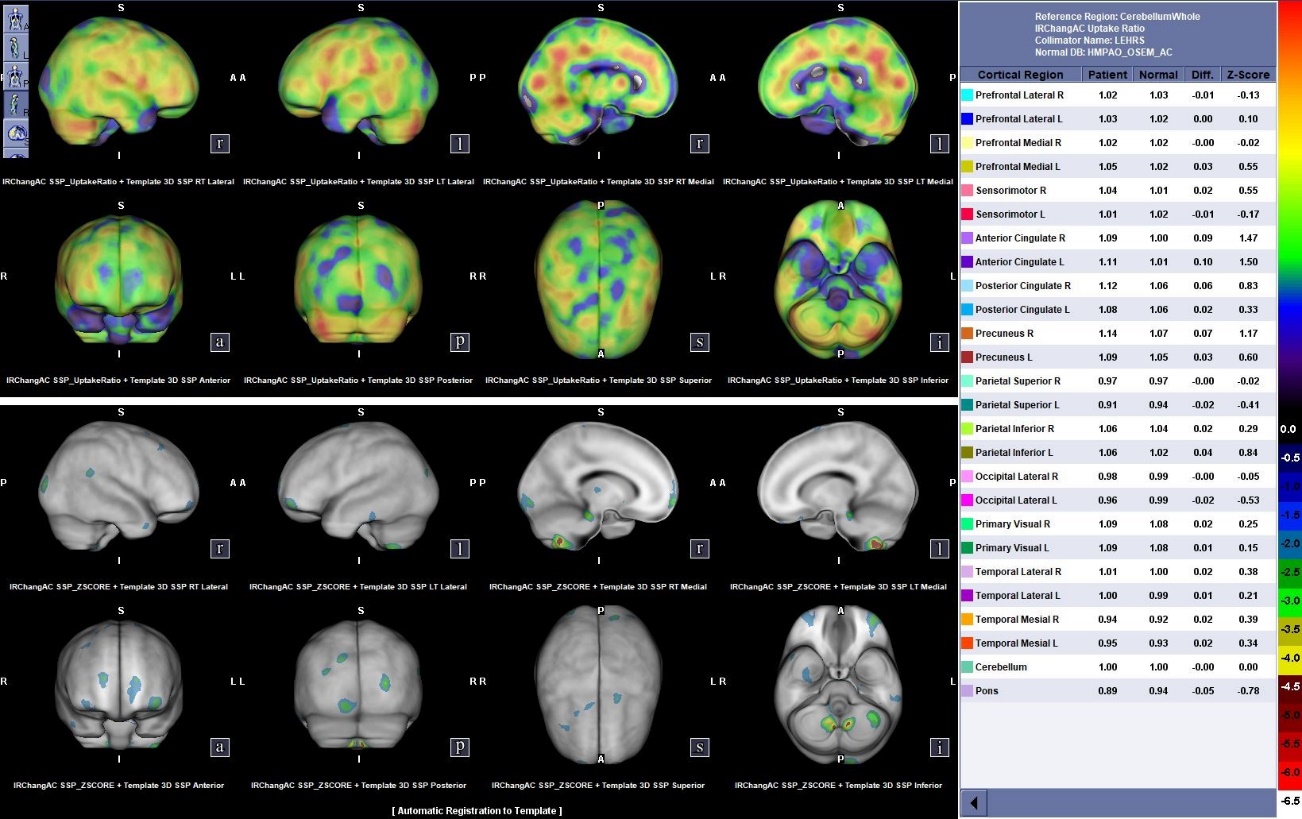


**Supplementary Figure 2: Study Flowchart**

Assessed for eligibility by 7^th^ Augst 2023

(*n* = 440)

Excluded (n = 384)

- No brain SPECT (*n* = 378)
- Missing data (*n* = 6)

Included (n = 56)

Identification

Inclusion

Analyzed

Analyzed (n = 56)
